# The Effect of Calcium, Citrate, and Urea on the Stability of Ultra-High Temperature Treated Milk: A Full Factorial Designed Study

**DOI:** 10.3390/foods8090418

**Published:** 2019-09-17

**Authors:** Maria A. Karlsson, Åse Lundh, Fredrik Innings, Annika Höjer, Malin Wikström, Maud Langton

**Affiliations:** 1 Department of Molecular Sciences, Swedish University of Agricultural Sciences, P.O. 7015, 75007 Uppsala, Sweden; ase.lundh@slu.se (Å.L.); maud.langton@slu.se (M.L.); 2 Tetra Pak Processing Systems AB, Ruben Rausings Gata, 22186 Lund, Sweden; fredrik.innings@tetrapak.com; 3 Norrmejerier Ek. Förening, Mejerivägen 2, 90622 Umeå, Sweden; annika.hojer@norrmejerier.se (A.H.); malin.wikstrom@norrmejerier.se (M.W.)

**Keywords:** UHT milk, interaction effects, calcium, citrate, urea, storage temperature, storage time, shelf-life

## Abstract

The composition of raw milk is important for the stability of dairy products with a long shelf-life. Based on known historical changes in raw milk composition, the aim of this study was to get a better understanding of how possible future variations in milk composition may affect the stability of dairy products. The effects of elevated calcium, citrate, and urea levels on the stability of ultra-high temperature (UHT) treated milk stored for 52 weeks at 4, 20, 30, and 37 °C were investigated by a two-level full factorial designed study with fat separation, fat adhesion, sedimentation, color, pH, ethanol stability, and heat coagulation time as response variables. The results showed that elevated level of calcium lowered the pH, resulting in sedimentation and significantly decreased stability. Elevated level of citrate was associated with color, but the stability was not improved compared to the reference UHT milk. Elevated levels of urea or interaction terms had little effect on the stability of UHT milk. Storage conditions significantly affected the stability. In conclusion, to continue produce dairy products with high stability, the dairy industry should make sure the calcium content of raw milk is not too high and that storage of the final product is appropriate.

## 1. Introduction

The composition and properties of raw milk are important for the manufacture of ultra-high temperature (UHT) treated milk to ensure a final product with high stability during storage, meeting the expected shelf-life. In the UHT process, milk is subject to high temperatures, above 135 °C for a few seconds, a treatment which may induce changes in the stability of the milk. Low stability can lead to fat separation, sedimentation, gelation, and browning [1]. Parameters considered to have most influence on the stability of UHT milk include protein composition, ionic calcium content, pH and parameters affecting all of these [2]. 

Calcium is important for the internal structure and stability of the casein micelle, both via the colloidal calcium phosphate associated to casein molecules and by the formation of calcium bridges between negatively charged residues of the caseins [3,4]. In milk serum, calcium can form complexes with other agents, or exists as free ions. Total calcium content in milk has been reported at average levels of 26–32 mM [5]. The calcium equilibria between the colloidal and serum phase is affected by pH, and a reduction in pH will increase the calcium ion concentration in the milk serum [3,6]. Also, temperature affects the calcium equilibria. When milk is stored at refrigerated temperatures, calcium phosphate will dissolve, whereas at higher temperatures, calcium phosphate will migrate into the micelle [7]. Previous studies comparing different calcium salts, i.e., calcium chloride, calcium lactate, and calcium gluconate, found that the effect on pH, heat coagulation time, and ethanol stability varied depending on the type of salt used [8,9]. 

Citrate, mainly present in milk serum, plays an important role in the mineral equilibria of milk and is part of a buffering system between calcium and hydrogen ions. Citrate improves the stability of the milk by forming soluble complexes with calcium, preventing precipitation of calcium phosphate [10]. High levels of citrate have been shown to increase calcium levels in milk serum and consequently a depletion of the colloidal calcium in the casein micelles [4]. The average value of citrate in milk is 7–11 mM [5], varying significantly with feed and stage of lactation [11,12].

Milk urea is known to vary with stage of lactation, season, and feed [13,14,15]. A higher concentration of urea acts as a stabilizing agent on the micelle [16]. It has been suggested that elevated levels of urea prevent crosslinking and aggregation of proteins, and also keep the pH stable [2]. It is suggested that at pH ≥6.8 urea, degraded to ammonia, has a buffering and stabilizing effect on milk protein concentrate suspensions, observed as longer heat coagulation time [17]. An average urea level of 4.7 mM in Swedish milk was reported by Lindmark-Månsson [18].

The stability of the milk can be evaluated by sensory attributes, including fat separation, fat adhesion to the packaging material, sediment formation, and color, varying with storage time and temperature [19]. During storage of UHT milk, fat globules float to the top, resulting in fat separation and fat adhesion [1]. Fat separation is closely correlated to and will increase with fat content, storage temperature, and milk fat globule size [20,21]. In UHT products, sediment formation is also a well-known problem. It is suggested that sediment consists of aggregates of proteins or protein particles of various sizes [22]. Casein micelles can, by the influence of gravity, sediment under their own weight [23]. Aggregation of micelles will further increase the rate of sedimentation [23]. Sediment formation has also been shown to increase with storage temperature [1,22]. During storage change in the color of UHT milk is mainly affected by the Maillard reaction, resulting in the formation of brown pigments in milk stored at warm temperatures [24].

To predict the stability of the resulting UHT milk, various tests have been used to assess the suitability of the raw milk for UHT processing, e.g. pH, ethanol stability, and heat coagulation time (HCT). Milk has a natural pH around 6.7, and lowering the pH will reduce the net charge on proteins and promote protein-protein interactions [25]. According to manufacturers of UHT processing equipment, raw milk should have a pH above 6.65 to be suitable for UHT processing [26]. Another commonly applied method for detection of poor quality milk is by evaluating its ethanol stability, which is a simple, cheap, and quick pass-fail-test [27]. It is recommended that milk for UHT processing should have an ethanol stability of 74% or higher [28]. Tsioulpas et al. [3] found that milk samples with low free calcium ion concentrations had consistently high ethanol stability, and it has been suggested that factors reducing the negative net charge of the casein micelle may reduce ethanol stability. The heat stability of milk is another commonly used predictive test of raw milk aimed at UHT processing. It is determined by the HCT, i.e., the time it takes for milk to visually coagulate when heated to temperatures above 100 °C [29]. Raw milk with poor heat stability is not suitable for UHT processing, due to increased fouling and sediment formation [2]. The heat stability of milk has been shown to be highly dependent on pH, with maximum heat stability around pH 6.65–6.70 [8].

The composition of milk delivered to Swedish dairies was previously investigated in 2001, and again in 2009 [18,30]. Comparing the composition of raw milk from 2009 with the composition reported in earlier studies, several changes that can be expected to have a negative impact on the stability of UHT milk were observed, for example, the calcium content had increased by 5%, whereas citrate and urea had decreased by 20% and 7%, respectively. Therefore, to get a better understanding of future scenarios, a study was designed to evaluate the impact of levels of calcium, citrate, and urea and their interaction effects on the stability of UHT milk during one year of storage at 4, 20, 30, and 37 °C. The study was set up as a full factorial experimental design, using fat separation, fat adhesion, sediment formation, color, pH, ethanol stability, and heat coagulation time as indicators of stability during storage.

## 2. Materials and Methods 

### 2.1. Design, Sample Preparation, Handling, and Storage

The experiment was set up as a 2^3^ full factorial design. In total nine batches of milk were prepared, at two production occasions, with elevated levels of calcium, citrate, and urea, including two unmodified reference batches (Table 1). Milk containing 20% higher concentrations of calcium, citrate, and urea were prepared based on previously reported average levels of 32, 9, and 4.7 mM of calcium, citrate, and urea, respectively [5,18]. Calcium (CaCl_2_·2H_2_O, VWR Chemicals, Leuwen, Belgium), citrate (Na_3_C_6_H_5_O_7_·2H_2_O, VWR Chemicals, Leuwen, Belgium), and urea (CO(NH_2_)^2^, Alfa Aesar, Karlsruhe, Germany) were dissolved in water and added to batches of 200 L pasteurized, standardized (1.5% fat), and homogenized milk to obtain calculated final concentrations of 38, 11, and 5.6 mM, in the milk. The modified milk was stored refrigerated overnight and subjected to UHT processing the following day. UHT treatment was conducted at the Tetra Pak Product Development Centre in Lund, Sweden, using upstream homogenization (150 + 30 bar), followed by indirect tubular heat exchangers at 137 °C for 4 seconds, and aseptically packed in 250 mL Tetra Brik Aseptic (TBA) packages. The UHT milk was transported at ambient temperature to the Swedish University of Agricultural Sciences (SLU), Uppsala, analyzed the day after delivery to SLU, so that the UHT milk was not older than 5 days post-production and thereafter stored at 4, 20, 30, and 37 °C for up to 52 weeks after production. During storage, new packages with milk were opened and analyzed every third week. Before the evaluation, samples were stored at room temperature overnight and analyzed the following morning. All measurements were done at ambient temperature unless otherwise stated.

### 2.2. Fat Separation, Fat Adhesion, Sediment Formation, and Color

Fat separation, fat adhesion, sediment formation, and color were measured as described in Karlsson et al. [19]. Fat separation was defined as the thickness of the cream layer floating on the surface and rated on a four-graded scale, i.e., no visual cream layer, waves of cream, a surface completely covered with fat or lumps/clots of fat. Fat adhesion, defined as the thickness and amount of fat adhering to the inside of the package after the milk was poured out, was compared with reference photos and rated on a scale of 0–4, modified from a protocol by the New Zealand Dairy Industry [31]. The amount of sediment formed at the bottom of the package was visually estimated and compared with reference photos, and graded on a scale 0–100%, where 0 corresponded to no sediment and 100 corresponded to the bottom of the package being completely covered by sediment [32]. Milk with sediment covering more than 45% of the bottom of the package was regarded as not acceptable for consumption. The CIELAB color space was measured with a CM-600d spectrophotometer (Konica Minolta, Shanghai, China). Using this technology, L* indicates lightness ranging from 0–100, a* indicates a range from green to red (−60 to +60), and b* a range from blue to yellow (−60 to +60). Milk with L* values under 76 was considered not acceptable for consumption. 

### 2.3. pH, Ethanol Stability, and Heat Coagulation Time

The pH, ethanol stability, and HCT were measured as described in Karlsson et al. [33]. In brief, pH was measured using an IoLine electrode (SI Analytics^®^, Mainz, Germany). Ethanol stability was defined as the highest ethanol concentration added to the sample without causing visual coagulation of the milk when equal volumes of milk and ethanol, at ethanol concentrations ranging between 40 and 100% in 2% increments, were mixed and incubated for 30 min [3]. HCT was defined as the time needed for visual coagulation of 0.5 mL milk in a sealed test tube whilst being rocked at 130 °C [29] using the dedicated equipment from Hettich Benelux (Geldermalsen, Netherlands). 

### 2.4. Statistical Analysis

Partial least squares regression (PLS) analysis was carried out using MODDE 11 software (Umetrics, Umeå, Sweden) with the factors calcium, citrate, and urea and the interaction terms calcium*citrate, calcium*urea, citrate*urea, and calcium*citrate*urea. Responses were fat separation, fat adhesion, sedimentation, color, pH, ethanol stability, and HCT. The first four principal components of the PLS model were fitted. Data were normalized by dividing the coefficients with the standard deviation of their respective response. Considering the known strong impact of storage temperature and storage time on stability, how the factors vary with storage temperature and storage time was also studied in the statistical evaluation.

## 3. Results and Discussion

### 3.1. Partial Least Squares Regression

**Figure 1:**
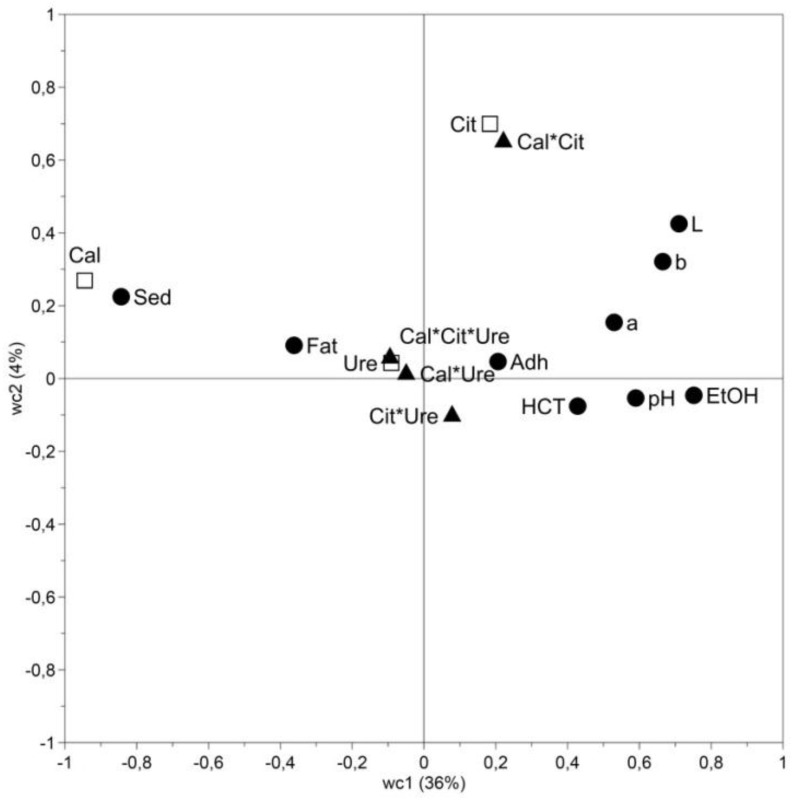
Partial least squares (PLS) for ultra-high temperature treated milk stored for 0-52 weeks at 4, 20, 30, and 37 °C. (□) factors, (▲) interaction terms, and (●) responses. The systematic variation in the data is to 36% and 4%, explained by the first and second principal components, respectively. Abbreviations: a = a*; Adh = fat adhesion; b = b*; Cal = calcium; Cit = citrate; EtOH = ethanol stability; Fat = fat separation; HCT = heat coagulation time; L = L*; Sed = sediment formation.

The systematic variation in the first principal component can be explained by calcium’s main effect on all factors, positively correlated to sediment formation and fat separation, and negatively correlated to all other factors (Figure 1 and Table 2). Citrate and the interaction between calcium and citrate, explaining the variation in the second principal component, were found to mainly affect the color (Figure 1 and Table 2). Urea and the interaction terms, except calcium and citrate, had no effect on the factors included in this study and were to be found at the center of the PLS plot (Figure 1 and Table 2). The four first principal components in total only explained 42% of the systematic variation and the constants of the regression coefficients were high (Table 2), indicating that additional variables, not included in the PLS model, could explain the variation in the data. For example, as discussed below, it is known that storage time and storage temperature affect the stability of UHT milk.

### 3.2. Effect of Calcium

**Figure 2:**
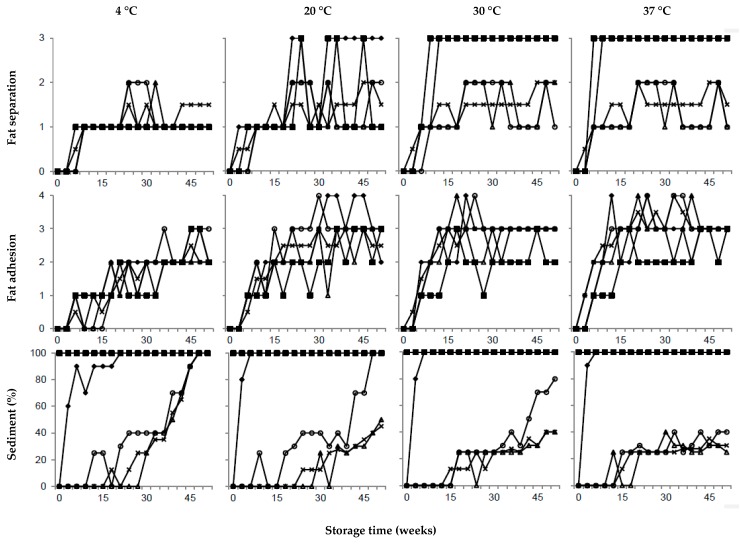
Variation in sediment formation, fat separation, and fat adhesion in ultra-high temperature treated milk for selected samples with elevated levels of (■) calcium, (○) citrate, (∆) urea, (♦) calcium and citrate, and average values of two batches (×) unmodified reference ultra-high temperature treated milk during storage from 0–52 weeks at 4, 20, 30, and 37 °C.

**Figure 3:**
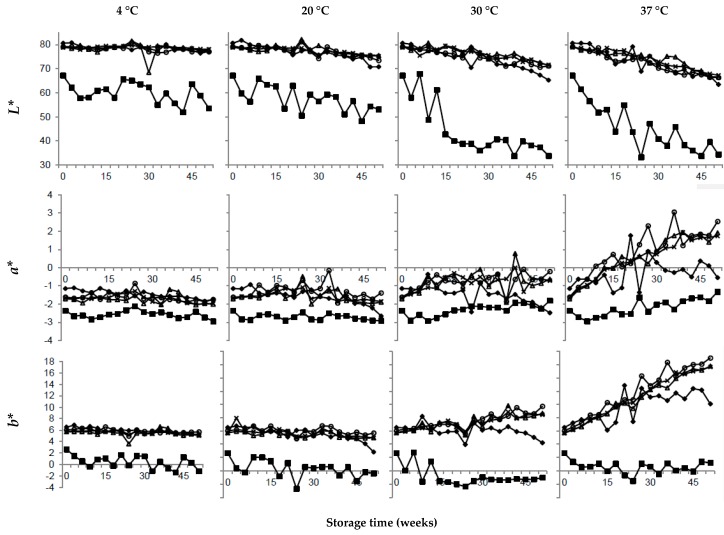
Variation in L* (lightness), a* (green-red), and b* (blue-yellow) in ultra-high temperature treated milk for selected samples with elevated levels of (■) calcium, (○) citrate, (∆) urea, (♦) calcium and citrate, and average values of two batches (×) unmodified reference ultra-high temperature treated milk during storage from 0–52 weeks at 4, 20, 30, and 37 °C.

**Figure 4:**
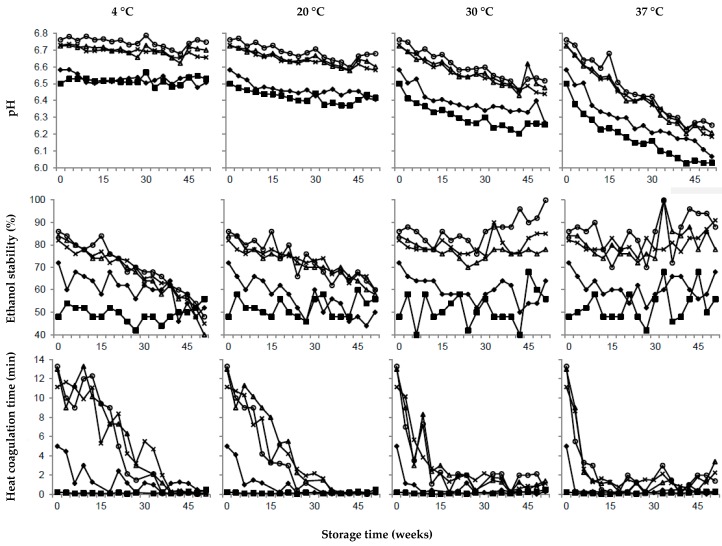
Variation in pH, ethanol stability, and heat coagulation time in ultra-high temperature treated milk for selected samples with elevated levels of (■) calcium, (○) citrate, (∆) urea, (♦) calcium and citrate, and average values of two batches (×) unmodified reference ultra-high temperature treated milk during storage from 0–52 weeks at 4, 20, 30, and 37 °C.

In this study, calcium had a significant effect on all responses, and elevated calcium content did, in multiple ways, give a less stable product compared to the reference UHT milk. Elevated calcium levels strongly correlated to sediment formation (Figure 1). Within a week after UHT processing, the entire bottom of the package was covered with sediment (Figure 2) and the product was thereby no longer acceptable for consumption, as the sediment covered >45% of the bottom of the package. Compared to the reference UHT milk, the L*, a*, and b* values were lower for UHT milk with high calcium content, giving a less white, less red, and less yellow product (Figure 3), likely due to fewer light scattering particles. In the same milk, the pH was 6.5 after UHT processing, remaining at this pH during storage at 4 °C, however, it decreased to 6.0 when stored for 52 weeks at 37 °C (Figure 4). Earlier studies have shown a correlation between sediment formation, color, and pH [34] and in agreement with our results, sediment formation was heavy, at a pH below 6.6 [35]. It is assumed that as pH is lowered, the calcium equilibria is shifted, leading to increased ionic calcium levels in serum and less colloidal calcium phosphate in the micelles, hence the casein micelles are destabilized, promoting aggregation of micelles, and sediment formation [36]. Consequently, the reflectance drops, due to a lower number of light scattering particles in milk serum. Also, in studies by Lewis et al. [6], an addition of 4.5 mM calcium chloride, corresponding to approximately 15% supplementation of calcium, resulted in large amounts of sediment at a pH of <6.6. Ramsey and Swartzel [1] and Malmgren et al. [22] found the amount of sediment to strongly depend on an increase of storage time and temperature. In contrast, our results do not clearly show an increase of sediment with increasing storage temperature. As seen in Figure 2, most sediment was formed in UHT milk stored at 4 °C, where the bottom of all packages were fully covered by sediment, corresponding to 100% sediment formation after 52 weeks of storage. In contrast, only 30% sediment was formed in the reference UHT milk when stored at 37 °C for 52 weeks (Figure 2). Our results agree with earlier studies by Wilson, Herreid, and Whitney [37], who also found more sediment formed in milk stored at 4 °C than at 21 and 38 °C. It has been suggested that different mechanisms are attributed to sediment formation at different temperatures [22,38]. The driving mechanism for casein micelle disintegration at low temperatures is the dissociation of β-casein. Whereas in UHT milk stored at high temperatures, sediment consists of large κ-casein depleted micelles [22,38].

In UHT milk with elevated calcium levels, ethanol stability was around 50% and HCT was less than a minute, independent of storage temperature and time. In comparison, it has been suggested that milk should have an ethanol stability of >74% to be suitable for UHT production [28]. In agreement with our results, Boumpa et al. [39] found a strong correlation between ethanol stability and ionic calcium. The addition of calcium is known to reduce the negative net charge of the casein micelles, resulting in lower ethanol stability. The effect of elevated levels of calcium on HCT seen in our study corresponds with results by Jeurnink and de Kruif [40], who reported that the HCT for pasteurized milk decreased from 16 to 10 minutes when the calcium content increased by 20%. In milk with high calcium activity, the electrostatic repulsion between micelles will be reduced and the stability will decrease, explaining the instant decline in HCT in UHT milk with elevated calcium content [40,41].

Previous studies have shown that different calcium salts have different effects on the stability of the milk [8,9], and that calcium chloride, which was used in this study, has a large negative impact on the stability of milk. The significant negative effect on the stability of milk by increasing the calcium content by 20% implies that lower levels of calcium chloride or other calcium salts should be used in future studies.

### 3.3. Effect of Citrate

When using a factorial design, it is possible to extract the main effects, comparing all values at high levels and low levels, and thereby increasing the statistical power of the analysis. Thus, comparing the average values of all four samples with elevated citrate content, i.e., also including the samples that in addition to elevated citrate content contained elevated contents of calcium and/or urea, regression coefficients showed that the factor citrate had a significant but small effect on the color of the UHT milk and was also correlated to fat adhesion, pH, and ethanol stability (Table 2). Compared to the reference UHT milk, the one UHT milk sample with elevated citrate content had a similar fat adhesion and color (Figure 2 and Figure 3), a slightly higher pH, and higher ethanol stability (Figure 4), and our results also show a tendency to more sediment formation (Figure 2). To reduce sediment formation in milk with high ionic calcium content, Gaur et al. [36] suggested the addition of citrate, as this increases the pH and chelates calcium. Zadow [34] showed that the addition of as little as 0.3% sodium citrate resulted in less sediment formation. Furthermore, less sediment gave a whiter skim milk, due to the remaining number of light scattering particles. The stabilizing effect of calcium chelators, e.g. citrate, has been shown to depend on calcium activity [42] and the pH [43] of the milk. Previous work has also shown that the addition of 5 mM citrate is required to have an effect on stability [43]. Consequently, in this study, elevating the citrate level by 2 mM did not improve the stability during storage compared to the unmodified reference UHT milk. It is therefore possible that the experimental design of our study, with only 2 levels of citrate, generated results that are somewhat misleading regarding the stabilizing effect of citrate.

### 3.4. Effect of Urea

In this study, the factor urea is located in the center of the PLS models (Figure 1), meaning that the factor had no effect on responses (Table 2). As seen in Figure 2 and Figure 3, the sensory attributes and, as in Figure 4, the stability during storage, were not improved compared to the reference UHT milk. Milk urea is known to vary with feed, and in a study by Reid et al. [15], cows with high crude protein intake had a significantly higher milk urea concentration compared to intake of a low protein feed. However no difference in heat coagulation time was reported [15]. In agreement with our results, Muir and Sweetsur [44] found that at a pH of 6.6–7.2, urea does not change the mechanism of the coagulation reaction, thus, in this pH region, addition of urea does not lead to a longer heat coagulation time or a higher ethanol stability. Earlier studies indicate that a level of >7 mM of urea is needed to improve stability [44]. In our study, urea was added to a final calculated concentration of 5.6 mM and compared to results from earlier studies, but this was too low to have an effect on the responses.

### 3.5. Effect of Interaction Terms

**Figure 5:**
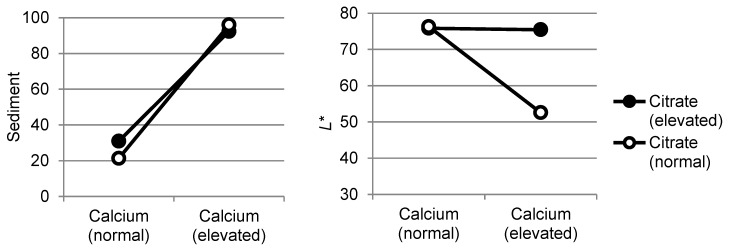
The interaction effects of calcium and citrate, for average values of all samples with normal and elevated levels, on sediment formation and L* (lightness) for ultra-high temperature treated milk stored for 0–52 weeks at 4, 20, 30, and 37 °C.

The interaction term calcium*citrate had a significant effect on sediment and color (Table 2). In our study, the combination of elevated levels of calcium and citrate had a short delay of few weeks on sediment formation during storage (Figure 2), but could not prevent heavy sediment formation. The actual effect of the calcium*citrate interaction on sediment formation could therefore be questioned. The regression coefficient was significant, but small, −0.05*** (Table 2) and the interaction effect shows almost parallel lines (Figure 5, left), which would indicate a low interaction. 

During storage, the L* values of UHT milk, with the combination of high calcium and citrate content, did not differ from L* values of the reference UHT milk (Figure 3). And, as seen in the interaction plot (Figure 5, right), elevated levels of calcium in combination with elevated levels of citrate did not give a reduction in lightness (L*). The interaction effect of calcium and citrate on L* (Figure 5) shows the same pattern as the interaction effect on a* and b* (results not shown), but with lower regression coefficients (Table 2). During storage at 4 and 20 °C, a* and b* values of UHT milk with high calcium and citrate content corresponded with values for the reference UHT milk. However, when stored at 30 and 37 °C, the increase in a* and b* that took place in the reference UHT milk were less pronounced, and less pigment was formed in UHT milk with elevated calcium and citrate content. This is possibly related to heavy sediment formation, leaving less particles in serum available for the Maillard reaction, hence less change in color during storage. All other interactions (calcium*urea, citrate*urea, and calcium*citrate*urea) had very little effect and are located at the centers of the PLS plots (Figure 1).

### 3.6. Effect of Storage Temperature and Storage Time 

Storage time and storage temperature were not part of the full factorial designed model to evaluate the effects of calcium, citrate, and urea. However, it is know that storage temperature and storage time affect the stability of UHT milk [19]. Our results show that independent of storage temperature, storage time was closely connected to fat separation and fat adhesion, i.e., the longer the storage, the more fat was floating on the surface and adhering to the package (Figure 2). In agreement with Ramsey & Swartzel [1], our study showed that as storage temperature increases, fat separation increases, as explained by Stoke’s law. In the reference UHT milk stored at 4 °C, formation of sediment was visible after 24 weeks of storage and thereafter increased linearly, at 52 weeks after production covering the entire bottom of the package (100%) (Figure 2). Sediment formation limited the shelf-life of the UHT milk stored at 4 °C to 40 week, thereafter >45% of the bottom of the package was covered, and the milk was no longer acceptable for consumption. At 20, 30, and 37 °C, at the end of the storage time, the reference milk had sediment covering 40% of the bottom of the package, never reaching an unacceptable level. 

The lightness (L*) of the reference UHT milk stored at 4 °C remained around 80 during the 52 weeks of storage (Figure 3), whereas when stored at 20, 30, and 37 °C, L* values decreased linearly to 75, 70, and 65, respectively. Changes in L* can be explained by less light scattering particles in the solution, due to fat separation and sediment formation. The PLS models (Figure 1) showed that the a* and b* values were correlated, hence a more red sample would also be more yellow. For the reference UHT milk stored at 4 and 20 °C, a* and b* values did not change during storage, whereas milk stored at 30 and 37 °C became considerably more brown (more red and yellow), corresponding to higher a* and b* values (Figure 3). At 37 °C, the change in color was most apparent, with a* and b* values increasing from −1 to 2 and from 6 to 16, respectively (Figure 3), eventually resulting in brown-colored milk. When UHT milk is stored warm, the Maillard reaction will contribute to the formation of brown pigment [45]. Our results corresponded with findings reported by Gaucher et al. [46] measuring b* values of around 15 in UHT milk stored at 40 °C for up to 26 weeks. 

In the reference UHT milk stored at 4 °C, the pH more or less remained at its initial value of 6.7 throughout the 52 weeks of storage (Figure 4). In contrast, at 37 °C, pH values decreased linearly by 0.5 units during storage from weeks 0 to 52. Our results for changes in pH corresponded with a study by Al-Saadi and Deeth [45], who stored UHT milk for 12 weeks at 5, 20, and 37 °C, and found the pH decreased from 6.6 to 6.5 when stored at 37 °C. Small changes by 0.1 pH have been reported to result in large changes in heat stability [2,40,41]. The Maillard reaction, including the development of formic acid, proceeds faster at a higher storage temperature, explaining the differences in pH between storage temperatures [45], as well as the rapid decrease in HCT during storage at elevated temperatures. 

In this study, after UHT treatment, the reference milk had an initial ethanol stability of >80% and an HCT of >10 min (Figure 4), decreasing during storage at 4 °C to around 50%, and when stored at 20 °C, went down to around 60%. In contrast, for UHT milk stored at 30 °C and 37 °C, we observed a tendency for increased ethanol stability during storage. It is known that during cold storage of milk calcium and phosphate, β-casein and other caseins will dissociate from the micelles into the milk serum, due to increased solubility at lower temperature and the weakening of hydrophobic bonds [5,41], resulting in reduced stability of the micelle, possibly explaining the decrease in ethanol stability and HCT during storage at 4 °C (Figure 4). In our study, the decrease in ethanol stability at 4 °C was not correlated to a reduction in pH (Figure 4). Neither was the decrease in pH during storage at 37 °C correlated to lower ethanol stability. In a recent publication, the visual coagulation in milk failing the ethanol stability test was believed to originate from a collapse of the outer hairy κ-casein layer of the casein micelle, causing the micelles to aggregate [25]. However, further studies are needed to fully understand the variation in ethanol stability during storage of UHT milk.

## 4. Conclusions

Calcium content, and the associated reduction in pH, had a significant negative effect on the stability of UHT milk and were strongly correlated to extensive sediment formation. Addition of citrate mainly affected the color of the UHT milk, and it is suggested that higher concentrations than those used in our study are needed to have a significant improvement on stability. The calcium*citrate interaction had a small but significant effect on sediment and color, whereas all other interactions, and urea, had no effect and thus did not affect the stability. Future research should be careful in designing experiments that involve milk’s colloidal stability and its related functionality in products. Measuring just a few levels of the major components that affect the colloidal stability of milk, like calcium, citrate and urea in this study, may exclude the critical combinations of the parameters. Regrettably, the data generated in this study give little new information about possible drivers for the aggregation phenomena behind sedimentation. Still, for the dairy industry to manufacture products of high quality and with high stability, monitoring the natural trends in milk composition, especially variations in calcium content, is important.

## Figures and Tables

**Table 1:** Experimental design of the 2^3^ full factorial study. Milk with elevated levels of calcium, citrate, and urea were prepared as below and the samples were subject to ultra-high temperature processing. Samples 1–5 and 6–9 were produced at different occasions, with one reference sample included at each occasion (samples 5 and 6). Abbreviations: +20% = elevated level; - = normal level.

Sample	Calcium	Citrate	Urea
1	+20%	-	-
2	-	+20%	-
3	-	-	+20%
4	+20%	+20%	+20%
5	-	-	-
6	-	-	-
7	+20%	+20%	-
8	+20%	-	+20%
9	-	+20%	+20%

**Table 2:** Significant regression coefficients relating to normalized variables for ultra-high temperature treated milk stored for 0–52 weeks at 4, 20, 30, and 37 °C. The size of the coefficient represents the change in response when a factor varies from normal to elevated level. *** *p* ≤ 0.001, and ** *p* ≤ 0.01.

	Constant	Factors			Interaction Terms			
		Calcium	Citrate	Urea	Cal*Cit	Cal*Urea	Cit*Urea	Cal*Cit*Urea
Fat separation	1.71 ***	0.39 ***						
Fat adhesion	2.16 ***	−0.18 ***	0.13 ***					
Sediment	1.38 ***	0.88 ***			−0.05 ***			
L*	5.90 ***	−0.61 ***	0.40 ***		0.47 ***			
a*	−1.12 ***	−0.49 ***	0.23 ***		0.20 ***			
b*	1.41 ***	−0.59 ***	0.37 ***		0.34 ***			
pH	36.53 ***	−0.61 ***	0.14 ***					
Ethanol stability	4.81 ***	−0.77 ***	0.23 ***					−0.07 **
Heat coagulation time	0.66 ***	−0.44 ***						
